# Addressing the COVID-19 pandemic challenges for operational adaptations of a cluster randomized controlled trial on dengue vector control in Malaysia

**DOI:** 10.1186/s12889-022-13026-x

**Published:** 2022-04-06

**Authors:** Mitra Saadatian-Elahi, Neal Alexander, Tim Möhlmann, Farah Diana Ariffin, Frederic Schmitt, Jason H. Richardson, Muriel Rabilloud, Nurulhusna Ab Hamid

**Affiliations:** 1 Service Hygiène, Epidémiologie, Infectiovigilance Et Prévention, Centre Hospitalier Edouard Herriot, Hospices Civils de Lyon, Lyon, France; 2 Public Health, Epidemiology and Evolutionary Ecology of Infectious Diseases, (PHE3ID) – Inserm - U1111 - UCBL Lyon 1 - CNRS – UMR5308 - ENS de Lyon, Lyon, France; 3 MRC International Statistics and Epidemiology Group, Department of Infectious Disease Epidemiology, London School of Hygiene and Tropical Medicine, Keppel St, London, WC1E 7HT UK; 4 In2Care B.V, Marijkeweg 22, 6709PG Wageningen, the Netherlands; 5 Medical Entomology Unit, WHO Collaborating Centre for Vectors, Institute for Medical Research, Ministry of Health Malaysia, National Institutes of Health, Block C, Jalan Setia Murni U13/52, Seksyen U13, Setia Alam, 40170 Shah Alam, Malaysia; 6 Bayer S.A.S, Environnemental Science, Crop Science Division, 16 rue Jean Marie Leclair, CS 90106 , 69266, Lyon Cedex 09, France; 7 Innovative Vector Control Consortium, Pembroke PlaceLiverpool, L3 5QA UK; 8 Université de Lyon, F-69000, Lyon, France; Université Lyon 1, F-69100, Villeurbanne, France; Hospices Civils de Lyon, Pôle Santé Publique, Service de Biostatistique Et Bioinformatique, F-69003, Lyon, France; CNRS, UMR 5558, Laboratoire de Biométrie Et Biologie Évolutive, Équipe Biostatistique-Santé, F-69100 Villeurbanne, France

**Keywords:** Dengue, Vector control, COVID-19, Malaysia, Randomized controlled trial

## Abstract

**Introduction:**

The COVID-19 pandemic placed an unprecedented overload on healthcare system globally. With all medical resources being dedicated to contain the spread of the disease, the pandemic may have impacted the burden of other infectious diseases such as dengue, particularly in countries endemic for dengue fever. Indeed, the co‐occurrence of COVID‐19 made dengue diagnosis challenging because of some shared clinical manifestations between the two pathogens. Furthermore, the sudden emergence and novelty of this global public health crisis has forced the suspension or slow-down of several research trials due to the lack of sufficient knowledge on how to handle the continuity of research trials during the pandemic. We report on challenges we have faced during the COVID-19 pandemic and measures that were implemented to continue the iDEM project (intervention for Dengue Epidemiology in Malaysia).

**Methods:**

This randomized controlled trial aims to assess the effectiveness of Integrated Vector Management (IVM) on the incidence of dengue in urban Malaysia by combining: targeted outdoor residual spraying (TORS), deployment of auto-dissemination devices (ADDs), and active community engagement (CE). Our operational activities started on February 10, 2020, a few weeks before the implementation of non-pharmaceutical interventions to contain the spread of COVID-19 in Malaysia.

**Results:**

The three main issues affecting the continuity of the trial were: ensuring the safety of field workers during the interventions; ensuring the planned turnover of TORS application and ADD deployment and services; and maintaining the CE activities as far as possible.

**Conclusions:**

Even though the pandemic has created monumental challenges, we ensured the safety of field workers by providing complete personal protective equipment and regular COVID-19 testing. Albeit with delay, we maintained the planned interval time between TORS application and ADDs services by overlapping the intervention cycles instead of having them in a sequential scheme. CE activities continued remotely through several channels (e.g., phone calls and text messages). Sustained efforts of the management team, significant involvement of the Malaysian Ministry of Health and a quick and smart adaptation of the trial organisation according to the pandemic situation were the main factors that allowed the successful continuation of our research.

**Trial registration:**

Trial registration number: ISRCTN-81915073. Date of registration: 17/04/2020, 'Retrospectively registered'.

## Background

Coronavirus disease 2019 (COVID-19), caused by severe acute respiratory syndrome coronavirus 2 (SARS‐CoV‐2), was declared as a pandemic on March 11, 2020. As of February 16, 2022, more than 400 million confirmed cases, including almost 6 million deaths have been reported to the World Health Organisation worldwide [1]. The pandemic led to an unprecedented overload of hospitals globally. In particular, tropical and subtropical regions endemic for dengue fever have had to bear a double burden on health care systems [2]. Indeed, the co‐occurrence of COVID‐19 increased hospital attendance, and hindered the diagnosis and management of all febrile diseases, including dengue, because of some shared clinical manifestation between the diseases [3, 4].

Malaysia, situated in South-east Asia, is a dengue-endemic country with an annual incidence rate that increased substantially from 82,851 cases in 2017 to 130,004 cases in 2019 [5]. In 2020 and 2021 respectively, a total of 90,304 and 26,365 dengue cases have been reported to the Malaysian Ministry of Health via the national dengue surveillance system; e-Dengue. The fear of contracting COVID-19 in healthcare facilities, cross-reactivity of the two pathogens on rapid tests [6], social distancing, and limited human movements related to the pandemic have been hypothesized to influence dengue incidence during the ongoing pandemic. These factors would tend to mitigate dengue incidence, or even decrease transmission, due to lockdown. On the other hand, a simulation modelling study found a spatial and location effect of lockdown on the increased number of dengue cases with a more pronounced effect in high mosquito abundance areas, and inside homes [7].

Recent research on the impact of lockdowns on dengue incidence showed greater number of dengue cases, despite a substantial decrease observed in the early stage of lockdowns in Malaysia [5, 8] and Singapore [9]. Another study reported similar findings in Thailand, but not in Malaysia and Singapore [10]. On the contrary, an overall 88% reduction in risk of dengue (RR 0.12; 95% CI: 0.08–0.17) during the period of mobility restriction was observed in Sri Lanka [11]. Dengue serotype switch [10], increase in time spent inside residence [8], disruption of dengue surveillance and vector control activities due to restricted human movements [12] and compliance with social distancing policies have been suggested as potential factors that could explain the observed findings.

The first local transmission of SARS-CoV-2 in Malaysia was detected on January 25, 2020 [13]. As of February 16, 2022, more than 3 million cases and 32,000 deaths have been reported in the country [14]. To contain the pandemic, the Malaysian government has implemented a range of non-pharmaceutical interventions, from country-wide lockdowns to different degrees of social distancing. The first Restriction of Movement Order (RMO) and later known as Movement Control Order (MCO) came into force on March 18, 2020 following the identification of COVID-19 cases subsequent to a religious event in Sri Petaling Selangor [8]. This was followed by several phases of MCO known as Conditional Movement Control Order (CMCO) and Recovery Movement Control Order (RMCO) until the end of May 2021. During the MCOs, only essential governmental and private services were allowed to operate, whereas the objectives of CMCO and RMCO were to relaunch the economy while respecting social distancing measures to effectively control the progression of the pandemic. Standard Operation Procedures (SOPs) and guidelines were shared by the Malaysian National Security Council through mass media (radio, television, newspapers), governmental websites, social media and text messages. They consisted of guidance that outlines health and safety measures while conducting any activities during the MCO. The SOPs were carefully crafted based on the avoidance of 3Cs (Crowded places, Confined spaces, Close conversation) and the practice of 3Ws (Wash hands, Wear masks, Warn against risks, symptoms, prevention and treatment) during the MCO enforcement.

Further to its impact on the burden of dengue or other infectious [15, 16] and non-communicable diseases [17–20], the COVID-19 pandemic has forced the suspension or slow-down of several research trials [21, 22]. This was mainly due to the sudden emergence and novelty of this global public health crisis, and lack of sufficient knowledge on how to handle the continuity of research trials while ensuring practical issues such as the security of both health care workers and patients during in-person consultations, shipment of the study products. The availability of health staff to continue running trials despite the added burden COVID-19 was also a major obstacle. We report on challenges we have faced and measures that were implemented to continue a randomized controlled trial for Dengue Epidemiology in Malaysia, during the COVID-19 pandemic and its related restriction of human movements.

## Main text

### Trial design

The intervention for Dengue Epidemiology in Malaysia (iDEM) project, is a cluster randomized controlled trial that aims to assess the effectiveness of Integrated Vector Management (IVM) on the incidence of dengue in urban Malaysia. A cluster is defined as one residential area composed of neighbouring buildings that share the same facilities such as parking lots, food court, groceries store, playground, and community halls.

The full trial protocol was published previously [23]. In short, this IVM strategy combines (1) targeted outdoor residual spraying (TORS) with K-Othrine Polyzone, (2) deployment of In2Care Mosquito Traps as auto-dissemination devices (ADDs), and (3) active community engagement (CE) activities. The latter is carried out by community engagement officers specially trained for the purpose of the trial, each responsible for a defined number of clusters.

Community engagement activities are held in management offices and community halls during weekdays, weekends or evening. They consist of meetings with the community in the study areas to explain the purpose of the study. In parallel, volunteers are identified to inform the field health care workers about potential vandalism, and the need for replacement of ovitraps or ADDs. Communities are urged to cooperate during spraying and not interfere with ADD equipment installed in their environment. We expect these regular meetings to build public understanding and trust that would favour continued compliance of participating clusters and their collaboration with the field workers during the intervention cycles. During CE visits, banners, posters and brochures are distributed to explain the objectives of the study and the role of the community during and following the deployment of the intervention.

In total, 280 clusters located in the Federal Territory of Kuala Lumpur and Putrajaya were allocated at random to receive either the combination of proactive IVM and routine vector control activities, or only the routine vector control activities.

**Fig. 1:**
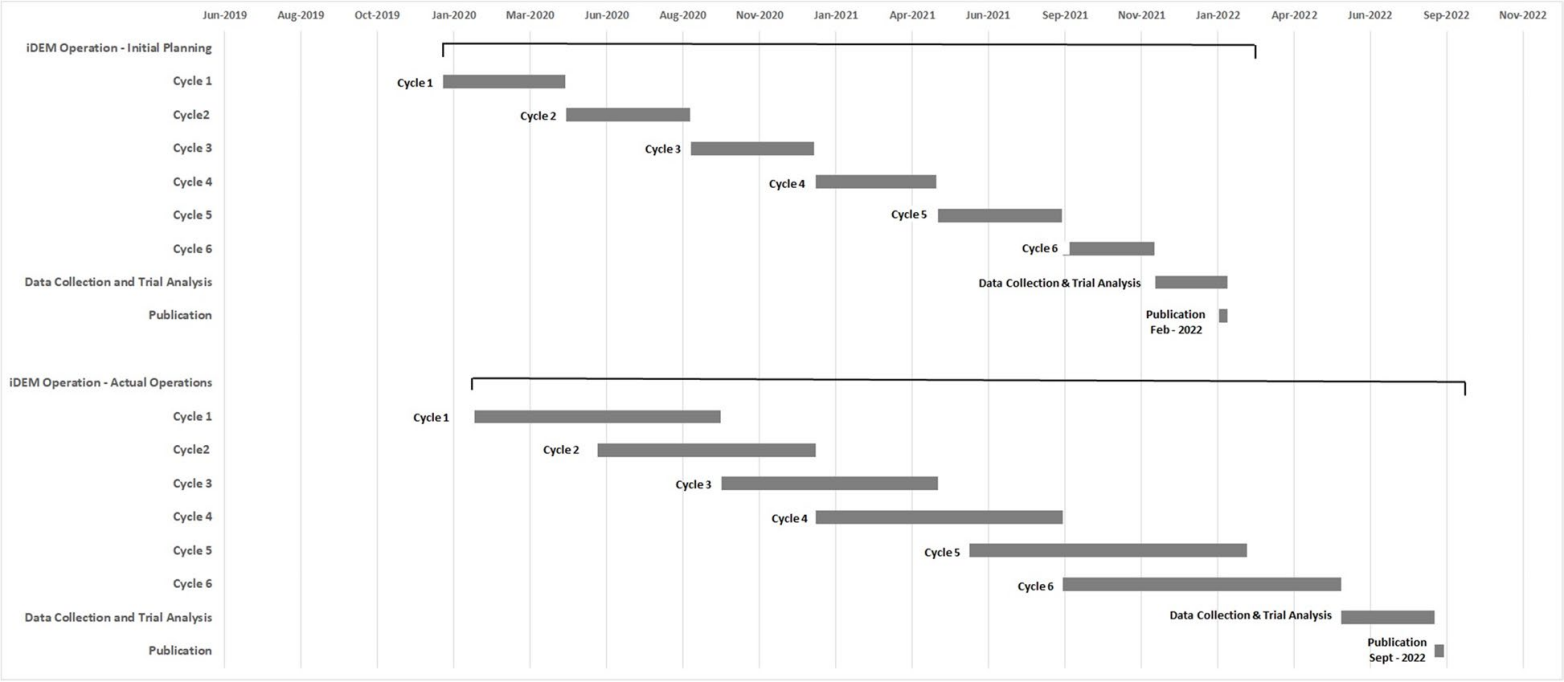
iDEM Project timelines before and after the start of the COVID-19 pandemic

The trial was expected to run for two years and include six intervention cycles. TORS was planned to be conducted every four months in all clusters in intervention areas. ADDs were distributed in the ground floors, first floors, top floors and evenly among intermediate floors at the beginning of the intervention and had to be serviced every two months. ADD services consist of ensuring that there is still adequate amount of the active ingredients, adding water inside the container, and recording and then replacing the damaged ADDs. Intervention cycles were planned to be sequential over the study period (Fig. 1). Community engagement events were planned as a continuous activity throughout the lifespan of the trial.

### Trial implementation

Our operational activities, including the implementation of TORS and ADDs, started on February 10, 2020, only a few weeks before the implementation of non-pharmaceutical interventions to contain the spread of COVID-19 in Malaysia. Due to the MCOs and the requirements to minimise face-to-face contacts with participants, the trial was completely stopped for a period of two weeks, from March 23 to April 5 2020, to prepare the documents required by the enforcement department (Malaysian Royal Police, Ministry of Health and Malaysia National Security Council). These documents were: a SOP for the community engagement steps during the pandemic; a SOP for operators and personnel to conduct TORS and quality control evaluations, and service the ADDs; and a SOP for personnel to collect the entomological data in the control and intervention clusters.

The pandemic forced the research and implementation team to think about how best to ensure the continuity of the trial and its procedures. The solutions had to be adaptable to different situations of the pandemic while preserving the safety of field workers, the continuity of vector control interventions and the CE activities.

Thanks to the strong support of the Institute for Medical Research (IMR), a division of the Malaysian Ministry of Health, an authorization letter was provided by the IMR director to the field operation team to declare the necessity of their work to continue the trial related vector control activities, i.e., ADD deployment and TORS.

### Major challenges and solutions deployed

The first issue was the safety of field workers when conducting their work during the pandemic, and this was ensured by equipping them with complete personal protective equipment (PPE) and by following the 3Ws and 3Cs rules. A formal training session was conducted in small groups on how to use and dispose of the PPE properly. Furthermore, field workers were tested for COVID-19 as soon as symptoms appeared or high-risk contacts were detected. Also, they benefited from vaccination once it was available in the country. All field workers, 9 research assistants, and 22 supervisors and operators have completed the two doses of COVID-19 vaccine.

The second most important issue was to adapt the implementation plan for vector control activities, i.e., to maintain the scheduled turnover for 6 cycles of TORS application every four months, and ADD services every two months [23]. Due to the pandemic and budget shortage, we had to consider the option of stopping the intervention after only 4 cycles. The trial protocol was written according to the SPIRIT statement, and no interim analysis or early stopping of the trial were anticipated. Also, publication and dissemination of the results based on only four cycles, instead of the six cycles initially planned, could have affected the power of the study and the credibility of the results. The maintenance of a two-year intervention trial was also critical in handling the high temporal variability of dengue. Thanks to operational changes adopted, we were able to maintain the original six cycles, as it is shown in the lower panel of Fig. 1.

During the MCO, vector control activities were carried out with a restricted number of field workers in each cluster. The field team had to be divided into small teams, each with 6 to 8 operators, rather than the original plan with up to 18–24 operators in a given cluster. The RMCO started on June 7 2020, allowing the field operation team to continue in a larger group but still comply with the aforementioned SOPs.

From January 2021 to May 2021, Malaysia experienced an increasing wave of COVID-19 cases throughout the country. As a response to the increasing cases, the government implemented another MCO following the same rules as the MCO 1.0. In three iDEM intervention clusters with a high number of COVID-19 cases, or under the instruction for Enhanced Movement Control Order (EMCO), ADDs services and entomological data collection activities were postponed for a period of four to six weeks until clearance was obtained from the Malaysia National Security Council. The obtention of the clearance was based on the number of positive COVID-19 cases in the area.

## Conclusions

Despite tremendous efforts of the team to maintain the field activities, the unexpected global pandemic impacted the field operations. The deployment of vector control activities and collection of entomological data were delayed due to the COVID-19 related restrictions. The number of clusters receiving TORS and ADD deployment per month was significantly reduced because only a small group of 6 to 8 operators could work together in a given cluster. This has led to the intervention cycles to be overlapping instead of being sequential, a delay of several months and significant increase in the trial budget, postponing the end of the trial to July 2022 instead of November 2021 originally planned (Fig. 1). Albeit with delay, the planned four-month interval between TORS application and ADDs services has been maintained. In particular, the four-month interval between each TORS application in the intervention clusters was crucial to preserve the residual efficacy of K-Othrine Polyzone, the product used for TORS [24, 25].

The budget gap was able to be filled i) by reducing the cost of human resources, and ii) by tremendous efforts of different members of the iDEM consortium to gather the extra funding.

CE is a crucial part of our proactive IVM and had to be well-maintained as much as possible. Our original CE plan was face-to-face meetings with volunteer inhabitants of each cluster to explain the purpose, objectives, procedure, and timelines of the iDEM trial, followed by a question-and-answer session. Before the implementation of the MCO, the CE team managed to meet with the management offices of all intervention clusters and obtained written informed consent to carry out the vector control activities after the briefing. organized community engagement talks were organized in seven of the 140 intervention clusters before the implementation of the MCO. During the MCO, face-to-face meetings were prohibited. However, CE activities continued remotely mainly through sending emails, phone calls, text messages, and letters informing the participants about activities of iDEM teams regarding the dates of TORS and ADD services. During the RMCO, the CE team could visit again the intervention and control clusters for face-to-face meeting but was limited to three clusters daily. Community talks could be conducted again but with smaller groups of less than 50 volunteer persons as compared to groups of up to 100 originally planned.

Previous experience suggests that mistrust and lack of understanding can be critical barriers to the continued compliance of residents and their collaboration with the field workers. In a study carried out in Johor Bahru, Southern Malaysia [26], we have shown that regular contact between the study population and the field workers created public trust and led to continuous compliance and collaboration during the intervention. As an example, residents contributed to a higher coverage of walls sprayed by TORS by removing bulky objects from the corridors at the time of interventions. During the early pandemic, limited face-to-face community engagement activities led to these bulky objects being removed less frequently, and more misuse and mistreatment of ADDS (e.g., being emptied of their contents, treat as a garbage bin). To mitigate this issue, ADDs were attached to the floor or to the walls, and extra stickers, explaining their function and last service date, were placed on the ADDs and nearby walls. In order to keep the residents informed and motivated, education and communication materials were left at post-boxes during the TORS activities by the field team.

The success of remote CE activities versus the original face-to-face meetings on the extent of public collaboration during iDEM vector control activities is hard to assess, as the pandemic occurred soon after the start of the trial. However, remote CE activities are likely to have been less engaging, leading to the kinds of consequences already mentioned, such as mistreatment of ADDs. On the other hand, vector control activities might have faced even more issues if we had not set up this alternative approach quickly. Thanks to remote CE activities, we maintained transmission of trial-related information to the public. Although we could not quantitatively compare the communication channels, we believe that text messaging was likely to have been among the most effective, due to their brevity, accessibility and high mobile phone coverage.

In addition to challenges presented so far, another issue doing a trial within such a pandemic context is the unpredictability of the extent of population behaviour change, and its epidemiological impact. However, we believe that, thanks to the randomized controlled design of the trial, any impacts of the pandemic on behaviour and dengue transmission would be similar in both intervention and control areas. Although the trial is not blinded, we think it unlikely that knowledge of the interventions would affect relevant behaviour. As a consequence, the magnitude of the effect of our intervention is unlikely to be affected by these parameters. A very important issue when evaluating the effectiveness of our intervention could be a reduction in the study power. Indeed, the sample size was calculated based on the incidence rates of the years 2017–2018. The actual incidence since then has been lower [27], and this could impact the study power to detect statistical significance between the two arms.

In conclusion, even though the pandemic has created monumental challenges which required additional efforts from the trial management and operations teams, we ensured the occupational safety of field workers, and we continued our trial, albeit with delay.

Sustained efforts of the management team, significant involvement of the Malaysian Ministry of Health and a quick and smart adaptation of the trial organisation according to the pandemic situation were the main success factors that allowed the continuation of our research that aims to improve knowledge of new tools to enhance protection of the population against dengue fever.

Sharing lessons learnt and best practices during this unprecedented global public health crisis may provide valuable insights that could be used in the near future to ensure the continuity of public health related research trials whilst ensuring field workers and participant safety.

## Data Availability

Not applicable as the paper is a description of experiences from the field.

## Abbreviations

RMO: Restriction Movement Control
MCO: Movement Control Order
CMCO: Conditional Movement Control Order
RMCO: Recovery Movement Control Order
EMCO: Enhanced Movement Control Order
SOP: Standard Operation Procedures
IVM: Integrated vector Management
TORS: Targeted Outdoor Residual Spraying
ADD: Autodissemination device
CE: Community Engagement
PPE: Personal Protective Equipment
